# Arterial Stiffness, Body Mass Index and Cardiovascular Disease Risk in Chinese Females at Various Ages

**DOI:** 10.31083/j.rcm2405144

**Published:** 2023-05-11

**Authors:** Lin Jin, Yichao Du, Mengjiao Zhang, Jianxiong Chen, Lei Sha, Mengmeng Cao, Lanyue Tong, Qingqing Chen, Cuiqin Shen, Lianfang Du, Dingqian Wang, Zhaojun Li

**Affiliations:** ^1^Department of Ultrasound, Guanghua Hospital Affiliated to Shanghai University of Traditional Chinese Medicine, 200052 Shanghai, China; ^2^Department of Neurosurgery, Shanghai General Hospital, Shanghai Jiao Tong University School of Medicine, 200080 Shanghai, China; ^3^Department of Medical Imaging, Weifang Medical University, 261053 Weifang, Shandong, China; ^4^Department of Ultrasound, Mindong Hospital Affiliated to Fujian Medical University, 355000 Ningde, Fujian, China; ^5^Department of Ultrasound, Shanghai General Hospital Jiading Branch, Shanghai Jiaotong University School of Medicine, 201812 Shanghai, China; ^6^Department of Ultrasound, Shanghai General Hospital, Shanghai Jiaotong University School of Medicine, 200080 Shanghai, China; ^7^School of Informatics, College of Science & Engineering, The University of Edinburgh, EH8 9AB Edinburgh, UK

**Keywords:** cardiovascular risk, arterial stiffness, women, aging, body mass index

## Abstract

**Background:**

This study investigated the correlation in parameters of arterial stiffness and cardiovascular disease (CVD) risk on age and body mass index (BMI) in Chinese females.

**Methods:**

This cross-sectional study enrolled 2220 females. Arterial stiffness was assessed by the measurement of arterial velocity pulse index (AVI) and arterial pressure volume index (API). Individual 10-year cardiovascular risk was calculated for each patient using the Framingham cardiovascular risk score (FCVRS).

**Results:**

API and AVI had a significant J-shaped relationship with age. Beginning at the age of 30 years, the API started to increase, while after 49 years, the increase in API was even steeper. AVI increased from the age of 32 years, and increased more rapidly after 56 years. The linear association between API and BMI following adjustment for age was significant (β = 0.324, 95% CI 0.247–0.400, *p *< 0.001). In the total study cohort, FCVRS scores increased by 0.16 scores for every 1 kg/$m^{2}$ increase in BMI and by 0.11 scores for each 1 value increase in API in the age adjusted model.

**Conclusions:**

API and BMI correlate with 10-year cardiovascular risk at various ages in females. Regardless of age, overweight females have a higher risk of increased API. Therefore API can be used for the early detection of CVD so that preventive therapy can be instituted in these high risk patients.

**Clinical Trial Registration:**

Registered on the official website of the China Clinical Trial Registration Center (20/08/2020, ChiCTR2000035937).

## 1. Introduction

Cardiovascular disease (CVD) remains the leading cause of morbidity and mortality among females worldwide [1]. According to European CVD statistics, CVD mortality was higher in females, especially in middle-income countries, and can be as high as 43% (vs 39% in males) [2].

Arterial stiffness is a marker of vascular damage and has been identified as an independent predictor of CVD [3, 4]. The traditional method of measuring arterial stiffness is pulse wave velocity (PWV), which represents the rate at which the pulse wave of circulating blood, travels to the peripheral vasculature and is proportional to the stiffness of the arterial wall and inversely proportional to the vessel diameter. However, it is more complicated to measure and its sensitivity is low [5]. The measurement of arterial velocity pulse index (AVI) and arterial pressure volume index (API) are validated approaches which can noninvasively quantify arterial stiffness [6]. Using oscillometric sensors within an upper arm blood pressure cuff inflated to suprasystolic pressure, a detailed analysis of the proximal brachial arterial pressure waveform can be acquired. Computational analyses of these waveforms are then used to obtain indirect measures of both central and systemic arterial stiffness. By quantitatively analyzing the peaks and troughs, AVI can be calculated, and API can be calculated by constructing a transmural pressure-volume characteristic curve [7].

AVI, reflecting the overall central artery stiffness, and API, reflecting the stiffness of peripheral arteries, are inexpensive techniques suitable for both large-scale epidemiological studies and clinical testing, with low operator dependence, making them attractive tools to assess cardiovascular health [8].

Obesity is associated with vascular remodeling and stiffness and has been shown to predict adverse CVD outcomes in both genders [9]. The negative effects of obesity on CVD health are higher in females compared to males [10]. Arterial stiffness is part of the arterial aging process, however; cardiovascular risk factors can accelerate and exacerbate arterial stiffness. In postmenopausal females, the incidence of CVD increases disproportionately [11]. Thus, gender-specific studies are needed to investigate the complex interactions between aging, body mass index (BMI), arterial stiffness and CVD risk to help identify patients at higher risk who would benefit from preventive treatment.

The aim of this study was to identify female-specific arterial stiffness risk factors for CVD, and the association of age and BMI to CVD risk.

## 2. Methods

### 2.1 Study Population

This single center, cross-sectional study population comprised 2220 females who visited the Health Management Center in the Shanghai General Hospital Jiading Branch, between August 2020 and December 2020. Approval for the study, No. 2021KY057, was issued by the Shanghai General Hospital Ethics Committee and registered on the official website of the China Clinical Trial Registration Center (20/08/2020, ChiCTR2000035937). All study subjects signed the informed consent form for this study. The study protocol was performed according to the Declaration of Helsinki. Patients undergoing hemodialysis or with atrial fibrillation, severe mental illness, pregnancy, acute illness, or with upper limb infections were excluded from the study, as were patients younger than 18 years of age.

### 2.2 Clinical Characteristics

All patients had a medical history, underwent a physical examination and anthropometric data collection. Age, height, weight, current medication, previous medical diagnoses, smoking history and alcohol consumption were recorded. BMI was calculated as weight (kg)/height ${\mathrm{(m)}}^{2}$.

Patients were stratified into three age groups according to the World Health Organization age criteria (18–44 years: young, 45–59 years: middle aged, ≥60 years: older) [12], and were stratified into three subgroups according to BMI (BMI <24 kg/$m^{2}$: normal, 24–28 kg/$m^{2}$: overweight, ≥28 kg/$m^{2}$: obese) [13]. Smoking was defined as a history of smoking for more than 1 year and smoking more than 1 cigarette per day on average.

### 2.3 Biochemistry

Collection of fasting venous blood samples for immediate processing and analysis at the time of the survey, included total cholesterol (TC), high-density lipoprotein cholesterol (HDL-C), low-density lipoprotein cholesterol (LDL-C), triglyceride (TG), fasting plasma glucose (FPG), albumin and uric acid (UA).

### 2.4 Measurement of Arterial Stiffness

AVI, API, systolic blood pressure (SBP), and diastolic blood pressure (DBP) were measured simultaneously based on a cuff oscillator (PASESA AVE-2000Pro, Shisei Datum, Tokyo, Japan). Smoking and caffeine were avoided for at least one hour before the examination. After resting for at least 5 minutes, the cuff was wrapped around the upper arm in the sitting position with the temperature controlled at 24–26 °C in a quiet room. API was calculated using the formula (X × 1/B), in which X is a constant and indicates the transverse wall pressure to be fitted. B is the coefficient of the fitting function, whose value was determined according to the algorithm for fitting the function to the transmural pressure-vascular volume characteristic curve [14]. AVI was calculated as A ×|V2|/|P1|, which is the ratio of the second valley to the first peak of the first-order differential waveform of the cuffed oscillation waveform, where A was taken as a constant 20 [14]. AVI and API became dimensionless indicators. Measurements were performed three times, and the average values of the assessments was calculated.

### 2.5 Framingham Cardiovascular Risk Score

The Framingham cardiovascular risk score (FCVRS) equation was used to estimate the 10-year CVD risk [15], including gender, age, smoking, TC, HDL-C, SBP hypertension and diabetes mellitus [16].

### 2.6 Statistical Analysis

For normally distributed values, the data were presented as mean and standard deviation for continuous variables and one-way Analysis of Variance (ANOVA) and post hoc least-significant difference (LSD) analysis was used to present the clinical characteristics, anthropometric measures, and biochemical parameters for each age group when significant. For non-normally distributed values, data were presented as median (along with first-third quartiles) and the Kruskal-Wallis and Wilcoxon signed-rank test was used to present the FCVRS score. For categorical variables, the data were presented as numbers and percentages by category for qualitative variables and the Chi-square test used for the descriptive analysis.

Pearson’s or Spearman correlation analysis was used to analyze risk factors for CVD with arterial stiffness and FCVRS. The relationship between age and AVI, and API were analyzed by restrictive cubic spline (RCS). Multivariable linear regression models were used to assess the association between API and FCVRS with adjustment for age and BMI. A two-tailed p value of less than 0.05 was considered statistically significant. Statistical analyses were performed using statistical tools package SPSS 23.0 (IBM, Armonk, NY, USA). RCS statistical analyses were performed using Stata 12 (Stata Corp, College Station, TX, USA).

## 3. Results

### 3.1 Baseline Characteristics

A total of 2220 females aged 20 to 79 years, with a mean age of 57 years, who had complete data sets were analyzed in this study. The cohort had an average BMI of 24.07 ± 3.65 kg/$m^{2}$, the brachial BP averaged 130/77 mmHg, API averaged 18 units (range 3–46 units), AVI averaged 29 units (range 4–66 units), and FCVRS averaged 11.42 (range –8–24). The study cohort included 29 current smokers (1.3%); 1160 patients (52.3%) had normal weight, 818 were overweight (36.8%), 242 were obese (10.9%). The SBP, DBP, TC, LDL-C and FPG in middle-aged and older patients were significantly higher than those in younger patients. The baseline characteristics of all study patients are shown in Table 1.

**Table 1.** **Basic characteristics of the study population (mean ± SD)**.

Variables		Total	18–44 years	45–59 years	≥60 years	*p*
n		2220	400	722	1098	
Age (years)		56.96 ± 12.59	35.97 ± 6.07	53.18 ± ${\mathrm{4.25}}^{*}$	67.08 ± 4.78^*#^	<0.001
Current smoker (%)		29 (1.3%)	4 (1.0%)	9 (1.2%)	16 (1.5%)	0.777
Anthropometrics						
	Height, cm	158.78 ± 5.18	160.33 ± 4.54	159.11 ± ${\mathrm{4.84}}^{*}$	158.00 ± 5.45^*#^	<0.001
	Weight, Kg	60.74 ± 10.04	60.52 ± 11.72	61.69 ± 9.72	60.20 ± ${\mathrm{9.54}}^{\#}$	0.007
	Body mass index, kg/$m^{2}$	24.07 ± 3.65	23.45 ± 3.97	24.34 ± ${\mathrm{3.36}}^{*}$	24.09 ± 3.50	0.002
	Systolic blood pressure, mmHg	129.86 ± 23.25	115.12 ± 17.44	128.08 ± ${\mathrm{20.44}}^{*}$	136.41 ± 24.17^*#^	<0.001
	Diastolic blood pressure, mmHg	77.16 ± 12.16	74.35 ± 11.28	78.93 ± ${\mathrm{11.92}}^{*}$	77.02 ± 12.42^*#^	<0.001
	Heart rate, beats/min	79.69 ± 12.34	84.49 ± 12.77	79.14 ± ${\mathrm{12.33}}^{*}$	78.31 ± ${\mathrm{11.75}}^{*}$	<0.001
Laboratory parameters						
	Uric acid, umol/L	286.27 ± 83.22	266.68 ± 71.61	281.30 ± ${\mathrm{79.78}}^{*}$	296.78 ± 87.77^*#^	<0.001
	Albumin, g/dL	43.10 ± 4.42	43.31 ± 4.42	43.61 ± 4.47	42.69 ± 4.35^*#^	<0.001
	Total cholesterol, mmol/L	4.61 ± 1.02	4.21 ± 0.89	4.79 ± ${\mathrm{0.991}}^{*}$	4.64 ± 1.03^*#^	<0.001
	Triglyceride, mmol/L	1.42 ± 0.94	1.16 ± 0.87	1.48 ± ${\mathrm{1.05}}^{*}$	1.48 ± ${\mathrm{0.86}}^{*}$	<0.001
	HDL cholesterol, mmol/L	1.22 ± 0.32	1.19 ± 0.29	1.23 ± ${\mathrm{0.31}}^{*}$	1.22 ± 0.33	0.076
	LDL cholesterol, mmol/L	2.86 ± 0.94	2.62 ± 0.85	3.03 ± ${\mathrm{0.91}}^{*}$	2.84 ± 0.98^*#^	<0.001
	Fasting plasma glucose, mmol/L	5.65 ± 1.53	5.11 ± 1.12	5.60 ± ${\mathrm{1.48}}^{*}$	5.88 ± 1.64^*#^	<0.001
Arterial stiffness parameters						
	AVI	17.96 ± 6.58	12.76 ± 4.38	17.98 ± ${\mathrm{6.15}}^{*}$	19.85 ± 6.51^*#^	<0.001
	API	29.41 ± 7.35	24.72 ± 5.29	27.98 ± ${\mathrm{6.32}}^{*}$	32.06 ± 7.51^*#^	<0.001
	FCVRS	13.00 (8.00, 16.00)	1.00 (–3.00, 4.00)	11.00 ${\mathrm{(9.00, 13.00)}}^{*}$	16.00 (14.00, 18.00)^*#^	<0.001

LDL, low density lipoprotein; HDL, high density lipoprotein; AVI, arterial velocity pulse index; API, arterial pressure volume index; FCVRS, Framingham cardiovascular risk score; SD, standard deviation. ^*^p < 0.05 vs the 18–44 years old group, ^#^ *p <* 0.05 vs the 45–59 years old group.

### 3.2 Age Distribution of Arterial Stiffness

**Fig. 1.**
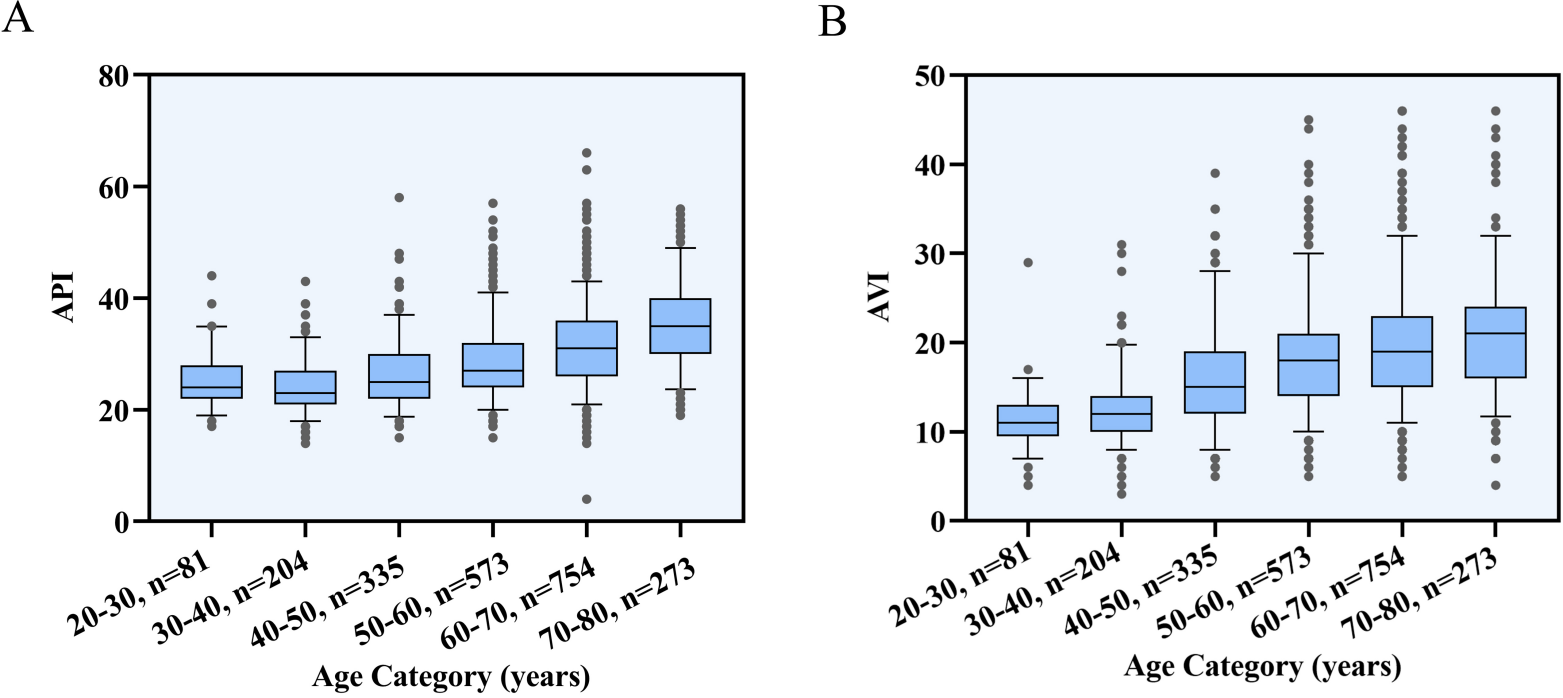
**Distribution of Mean API and AVI by Age**. (A) Box plots showing API by decades of age. (B) Box plots showing AVI by decades of age. The lower and upper limits of the box plots are the 5% and 95% percentiles; internal horizontal lines indicate medians; T‑bars represent the percentage of the 95% range; and the circles indicate outliers. AVI, arterial velocity pulse index; API, arterial pressure volume index.

**Fig. 2.**
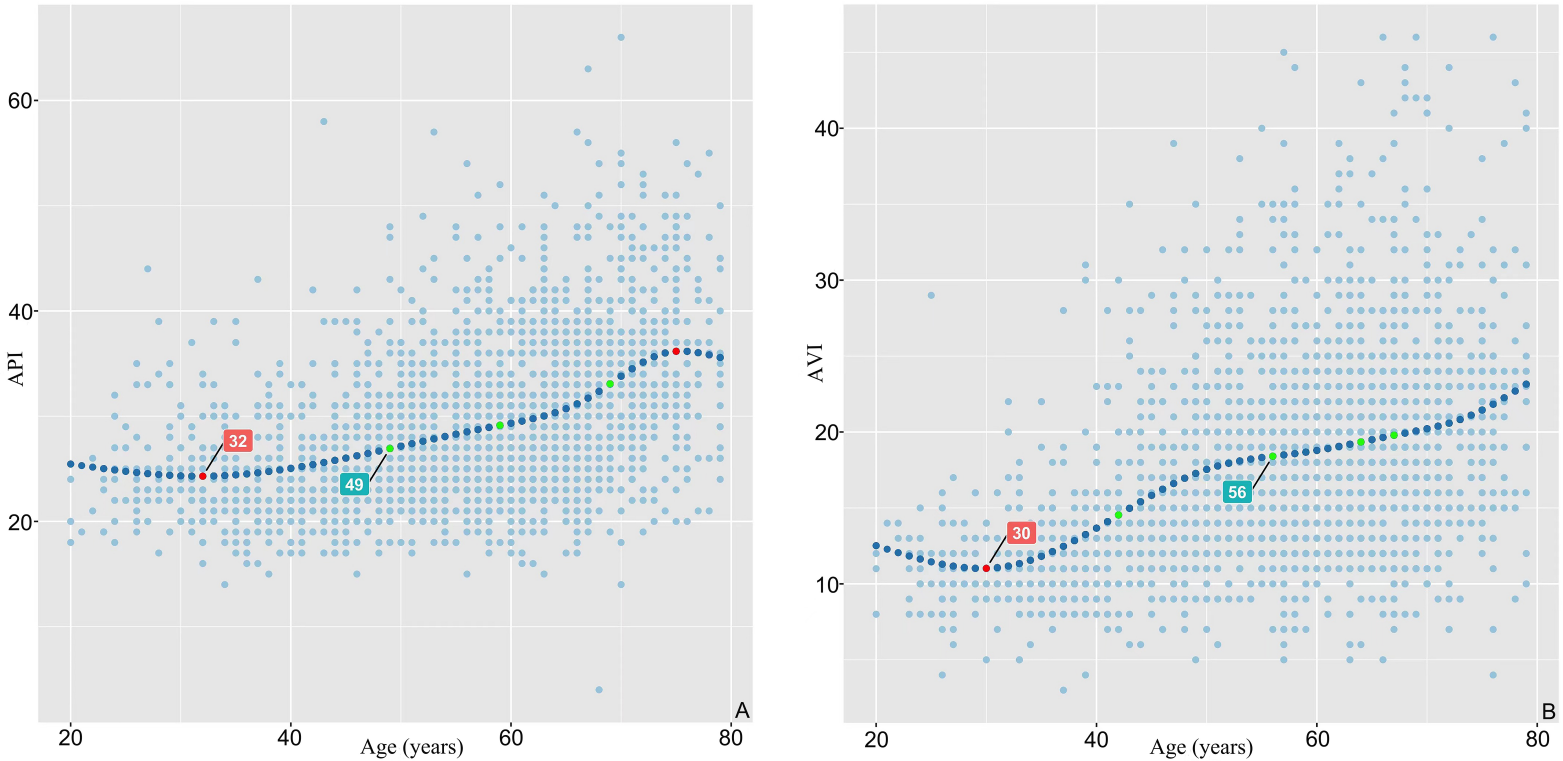
**Correlations between API, AVI and age based on Restricted Cubic Spline Functions**. (A) There was a significant J-shaped relationship between API and age. The age corresponding to the lowest API value was 30 years and when API increased rapidly, the corresponding age was 49 years. (B) There was a significant J-shaped relationship between AVI and age. The age corresponding to the lowest AVI value was 32 years, and when AVI increased rapidly, the corresponding age was 56 years. API, arterial pressure volume index; AVI, arterial velocity pulse index.

Arterial stiffness was assessed by AVI and API across the various age groups. Fig. 1 displays the absolute values and distributions of AVI and API for each age decade. Whereas AVI and API were low in young, it gradually increased and became more disperse with age. Middle aged and older females exhibited more vascular stiffness. API and AVI had different magnitudes of growth in different ages. Fig. 2 showed that there was a significant J-shaped relationship between AVI, API and age. Beginning at the age of 30 years, the API started to increase, while after 49 years the increase in API was even steeper. AVI increased from the age of 32 years, and then increased rapidly after the age of 56 years.

At all ages, API was low in patients with a normal BMI and it gradually increased in overweight and obese females who exhibited stiffer vessels than normal females. However, only overweight and obese young females had higher AVI (Table 2).

**Table 2.** **Arterial parameters (mean ± SD) grouped by age and BMI**.

Variables		Normal	Overweight	Obese	*p*
18–44 years					
	n	258	102	40	
	Systolic blood pressure, mmHg	110.90 ± 14.71	118.96 ± ${\mathrm{16.84}}^{*}$	132.53 ± 22.05^*#^	<0.001
	Diastolic blood pressure, mmHg	71.76 ± 9.74	76.97 ± ${\mathrm{11.98}}^{*}$	84.33 ± 11.86^*#^	<0.001
	AVI	12.09 ± 3.93	13.89 ± ${\mathrm{4.93}}^{*}$	14.25 ± ${\mathrm{4.75}}^{*}$	<0.001
	API	23.98 ± 5.07	25.17 ± ${\mathrm{5.32}}^{*}$	28.35 ± 5.13^*#^	<0.001
	FCVRS	0.00 (–5.00, 3.00)	2.00 ${\mathrm{(–2.00, 4.00)}}^{*}$	4.50 ${\mathrm{(0.00, 6.00)}}^{*}$	<0.001
45–59 years					
	n	343	304	75	
	Systolic blood pressure, mmHg	123.43 ± 19.34	130.78 ± ${\mathrm{20.56}}^{*}$	138.39 ± 19.25^*#^	<0.001
	Diastolic blood pressure, mmHg	76.17 ± 11.05	80.25 ± ${\mathrm{11.83}}^{*}$	86.17 ± 12.36^*#^	<0.001
	AVI	17.87 ± 6.40	18.15 ± 6.17	17.79 ± 4.79	0.809
	API	26.69 ± 5.48	28.97 ± ${\mathrm{6.87}}^{*}$	29.85 ± ${\mathrm{6.48}}^{*}$	<0.001
	FCVRS	11.00 (8.00, 12.00)	11.00 ${\mathrm{(9.00, 13.00)}}^{*}$	12.00 (11.00, 15.00)^*#^	<0.001
≥60 years					
	n	559	412	127	
	Systolic blood pressure, mmHg	132.17 ± 22.91	140.38 ± ${\mathrm{24.04}}^{*}$	142.17 ± 26.69^*#^	<0.001
	Diastolic blood pressure, mmHg	74.40 ± 11.61	79.02 ± ${\mathrm{12.34}}^{*}$	82.01 ± ${\mathrm{13.44}}^{*}$	<0.001
	AVI	20.21 ± 6.58	19.62 ± 6.18	18.91 ± 7.16	0.109
	API	30.84 ± 7.37	33.03 ± ${\mathrm{7.43}}^{*}$	34.37 ± ${\mathrm{7.41}}^{*}$	<0.001
	FCVRS	15.00 (13.00, 17.00)	16.00 ${\mathrm{(14.00, 18.00)}}^{*}$	16.00 (14.00, 18.00)	<0.001

AVI, arterial velocity pulse index; API, arterial pressure volume index; FCVRS, Framingham cardiovascular risk score; BMI, body mass index; SD, standard deviation. ^*^p < 0.05 vs the normal group. ^#^*p <* 0.05 vs the overweight group.

### 3.3 Association between Arterial Stiffness and Clinical Characteristics

**Fig. 3.**
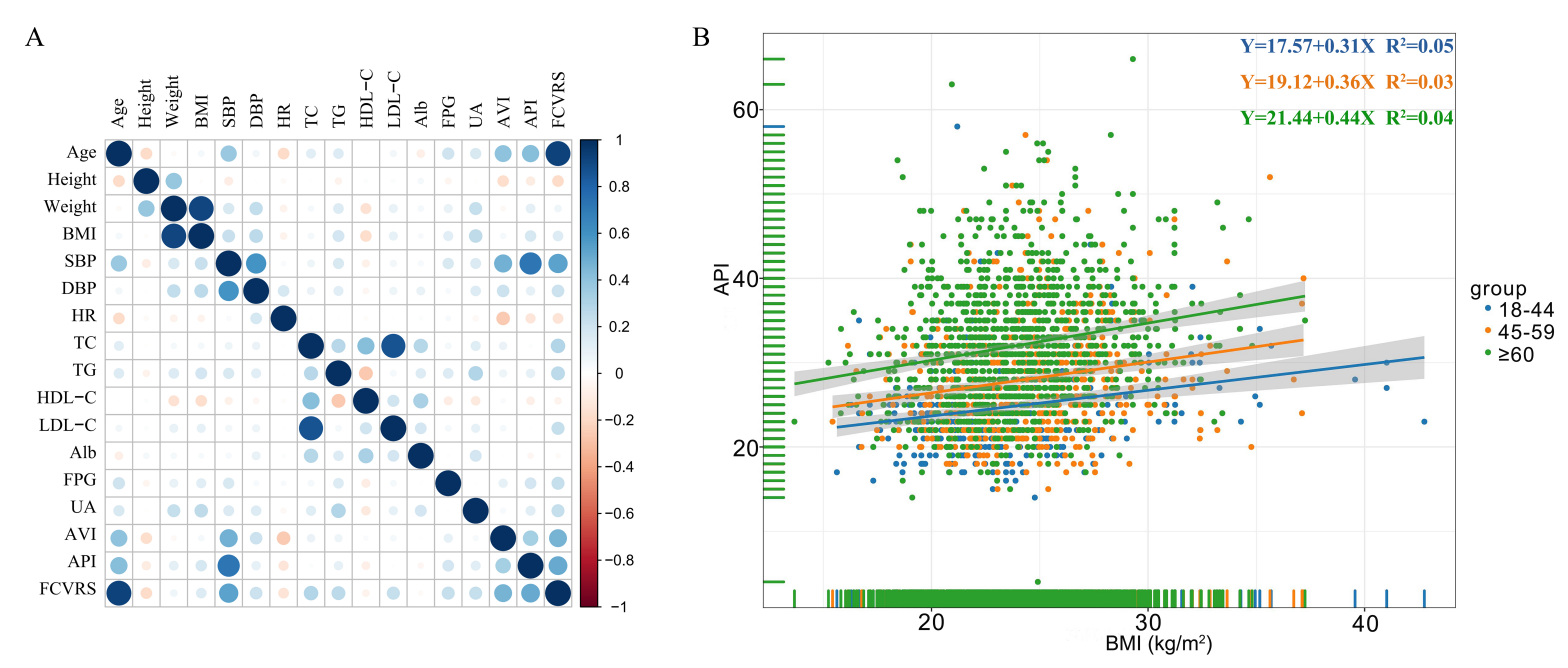
**Associations between arterial stiffness indices and clinical characteristics**. (A) Age, BMI, SBP, DBP, TG, FPG, FCVRS were positively correlated with AVI and API, and heart rate was inversely correlated with AVI and API. (B) BMI was significantly associated with API. BMI, body mass index; SBP, systolic blood pressure; DBP, diastolic blood pressure; HR heart rate; TC total cholesterol; TG triglyceride; LDL-C, low density lipoprotein cholesterol; HDL-C, high density lipoprotein cholesterol; FPG, fasting plasma glucose; UA, uric acid; Alb, albumin; AVI, arterial velocity pulse index; API, arterial pressure volume index; FCVRS, Framingham cardiovascular risk score.

Associations between arterial stiffness indices and clinical characteristics were summarized in Fig. 3. Positive correlations were observed for age, BMI, SBP, DBP, TG, FPG, FCVRS, and AVI and API. Heart rate was inversely correlated with AVI and API.

Linear regression analysis showed that BMI was significantly associated with API in the total study cohort (β = 0.324, 95% CI 0.247–0.400, *p *< 0.001) but not with AVI in the age adjusted model (Fig. 3).

### 3.4 Age Based Associations of API with FCVRS

**Fig. 4.**
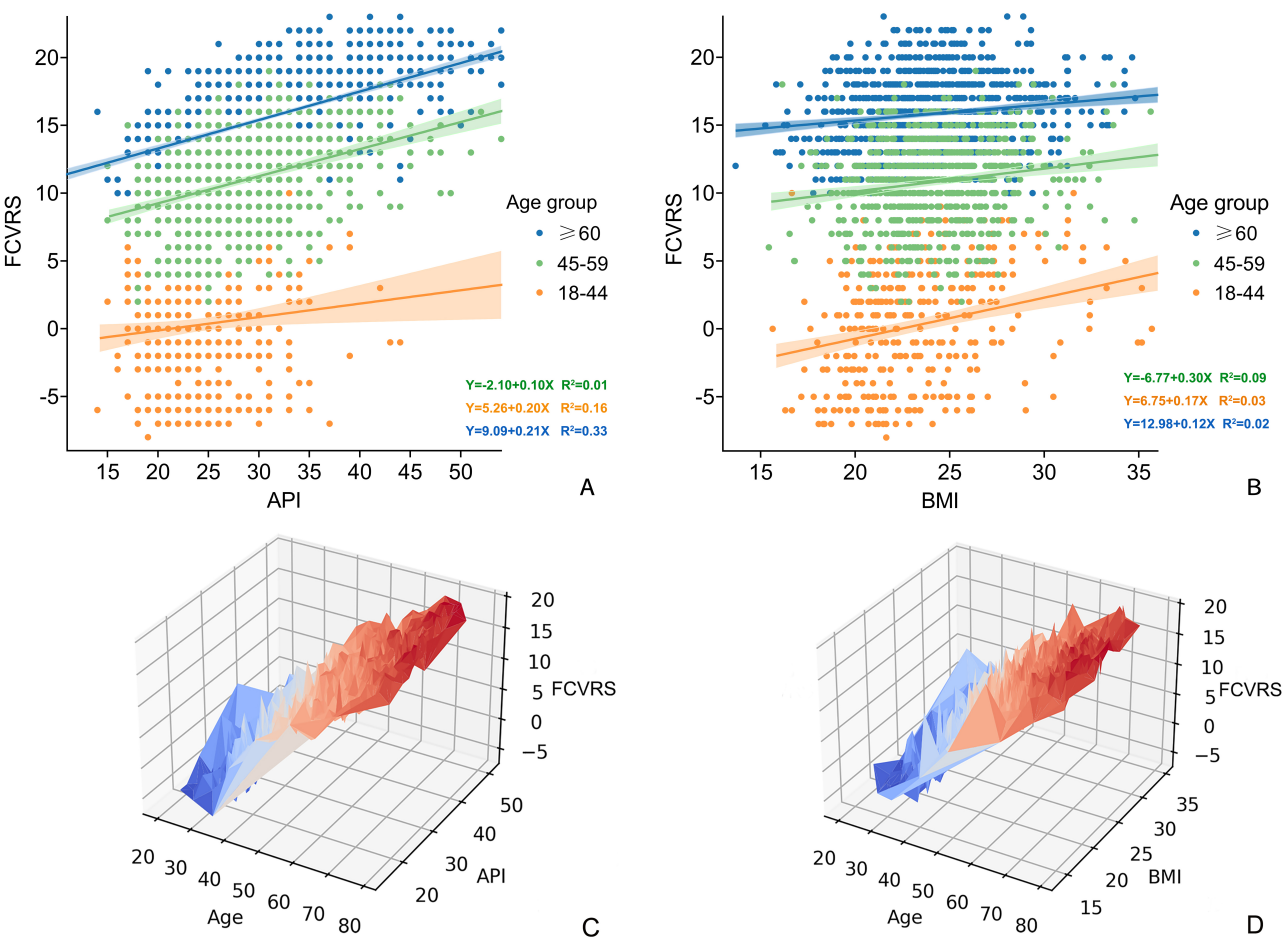
**Correlation between API, age, BMI and FCVRS**. (A) Associations between the Framingham Risk score and API by age groups. (B) Associations between the Framingham Risk score and BMI by age groups. (C) Age/API relations of the Framingham Risk score, age (x axis, years), and API (y axis, values) and FCVRS. (D) Age/BMI relationship of the Framingham Risk score, age (x axis, years), and BMI (y axis, kg/$m^{2}$) and FCVRS. Age, API/BMI and interaction with FCVRS are highly significant. API, arterial pressure volume index; BMI, body mass index; FCVRS, Framingham cardiovascular risk score.

The association between API, BMI and FCVRS was assessed using linear regression analysis, adjusted for age, and was significant in females. FCVRS scores increased by 0.16 scores for every 1 kg/$m^{2}$ increase in BMI and by 0.11 scores for each 1 value increase in API in the age adjusted model (**Supplementary Fig. 1**). In the age categories, the linear association between API and FCVRS remained significant (young group, r = 0.116, *p *< 0.05; middle aged group, r = 0.403, *p *< 0.001; older group, r = 0.571, *p *< 0.001), as well as BMI (young group, r = 0.296, *p *< 0.001; middle aged group, r = 0.181, *p *< 0.001; older group, r = 0.150, *p *< 0.001) (Fig. 4).

## 4. Discussion

This study evaluated the association of arterial stiffness and the Framingham 10-year cardiovascular risk score from adult to older females in a Chinese population, with a specific focus on age-BMI interactions. Arterial stiffness showed a substantial augmentation of the age-related increase in AVI and API in middle and old age, and both AVI and API had a significant J-shaped relationship with age. BMI was significantly associated with API but not with AVI in the age adjusted model. Overweight and obese females had a higher AVI compared to females with normal BMI only in the young group. These results demonstrate that excess BMI in females was associated with an increased risk of peripheral arteries stiffness. In addition, API, taken as an indicator of cardiovascular risk, was significantly associated with FCVRS. FCVRS scores increased by 0.16 scores for every 1 kg/$m^{2}$ increase in BMI and by 0.11 scores for each 1 value increase in API in the age adjusted model.

Artery stiffening is a manifestation of vascular aging, and has been recognized as an important cardiovascular risk factor [17, 18]. Previous studies [19] have shown that there are mechanistic differences in vascular aging and arterial stiffening between females and males. Age, obesity, hypertension and menopause are important determinants of aortic and peripheral arterial stiffness in the high cardiovascular risk female population [20]. In particular, the age-related increase in arterial stiffness was observed after menopause [21, 22, 23]. In this study, middle-aged and older females exhibited higher arterial stiffness. From the age of 30 years, the API started to increase, while after 49 years the increase in API was even steeper. AVI increased from the age of 32 years, and increased rapidly after the age of 56 years. Furthermore, API and AVI have different magnitudes of increase in middle and old age. The increase in AVI was most pronounced among females in their 50s and 60s, while API was significantly higher in the older age group after 60 years. Our results were consistent with previous studies, which found that elastic arteries became stiffer than peripheral muscular arteries in middle age due to degradation of elastin [20, 24]. In addition, hormonal changes, oxidative stress, as well as a higher susceptibility to conventional vascular risk factors accumulating after menopause might play a role in the regulation of compliance in larger arteries [25, 26, 27].

Our results are in agreement with previous studies [28], in which female peripheral muscular arteries were stiffer with increasing BMI from adulthood, with a linear increase in API with age. The strength of this correlation also depended on the age group; the older the participants, the stronger the dependence. However, AVI increased with BMI only at a young age. Previous studies have shown [6, 29] that these two variables, API and AVI, have different meaning in the assessment of target organ damage and that the risk factors associated with both are not the same. BMI is influenced by sex, age and race [30]. For older adults, increased arterial stiffness was more related to abdominal visceral fat distribution and adiposity than to increased BMI [31]. While in young adults, obese individuals had an increase in aortic stiffness, independent of BP level, race, and age [32]. Ferreira *et al*. [33] found that total trunk fat was adversely associated with large artery stiffness. However, the influence of body fat on arterial stiffness remains controversial. Tapolska *et al*. [34] mentioned that obesity itself lead to an increase in cardiac output and can affect arterial stiffness measurements, and that this issue required further research.

Our study demonstrated a significant correlation between API and BMI with FCVRS in females at various ages. The risk of CVD increased significantly in middle aged and older females, especially in those who were overweight. Previous studies suggest that female sex hormones protect against CVD until the ages of menopause, but this protection may be lost in obese patients [35]. In addition, obesity induced insulin resistance was associated with a greater increase in arterial stiffness, and is a stronger determinant of CVD risk in females [36, 37].

Finally, it is noteworthy that in female individuals with aging and obesity, increased aortic stiffness also may contribute to the development of CVD risk. Artery stiffness, measured with a simple clinical tool such as API, combined with BMI, remains a good predictor of CVD risk.

## 5. Limitations

A major strength of this study is that the large sample size allowed us to analyze the correlation between the new oscillometric indices of arterial stiffness and cardiovascular score in Chinese females and age–BMI interactions. This was a single center cross-sectional study, which represented the sample characteristics of Chinese Oriental females, and our results may not be generalizable to other populations or ethnic groups. Nevertheless, the arterial stiffness data in Chinese females have good validity and reliability. The sample size differed by age range which is a potential source of bias however the consistency of the results makes significant bias less likely. Moreover, our study samples were taken from the natural population, which may provide a reference standard for the future study of the control population. In addition, our study enrolled some individuals aged <30 years, and these individual CVD risk scores were calculated according to the FCVRS equation. Finally, we had no data on the body fat distribution or visceral fat adiposity of the female participants, which is a significant determinant of arterial stiffness, and we did not used other methods to detect vascular stiffness, such as ultrasound, which is a significant determinant of arterial stiffness [38, 39]. These techniques will be assessed in future studies.

## 6. Conclusions

API and BMI correlated with 10-year cardiovascular risk measured by the Framingham cardiovascular risk scores at various ages in females. Regardless of age, overweight females have a higher risk of higher API. Therefore, API can be used for early detection of injury to peripheral arteries, and can be helpful in the identification of individuals with different CVD risks and provide guidance for early preventative treatment for CVD.

## Data Availability

All data generated or used during the study appear in the submitted article.

## Supplementary Material

Supplementary material associated with this article can be found, in the online version, at https://doi.org/10.31083/j.rcm2405144.
