# Supplementary figures and images for: Arterial Stiffness, Body Mass Index and Cardiovascular Disease Risk in Chinese Females at Various Ages
Source: Rev Cardiovasc Med. 2023 May 11;24(5):144. doi: 10.31083/j.rcm2405144 (PMC11273021; doi:10.31083/j.rcm2405144)

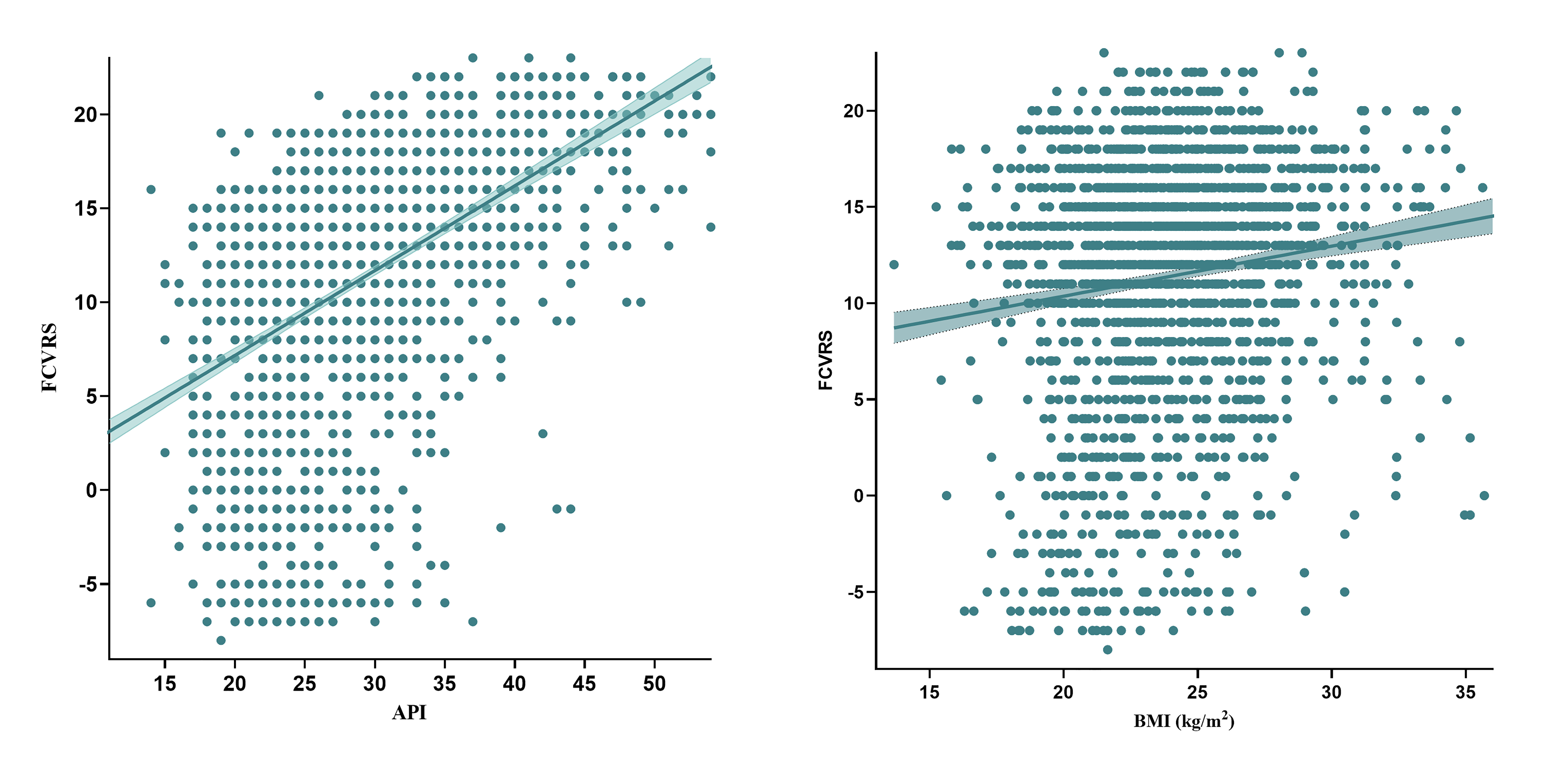

Supplement: Supplementary file 1 [file 2153-8174-24-5-144-s1.zip › 2153-8174-24-5-144-s1.tif]
